# Study of the effects of adapted Tango and multidimensional intervention in pREvention of dementia in agiNG: developing healTHy lifestyle programs (STRENGTH Project)—the experimental protocol of a prospective randomised controlled trial

**DOI:** 10.1007/s40520-020-01504-4

**Published:** 2020-03-02

**Authors:** Cinzia Giuli, Cristina Paoloni, Elpidio Santillo, Marta Balietti, Paolo Fabbietti, Demetrio Postacchini, Francesco Piacenza

**Affiliations:** 1 Geriatrics Operative Unit, Italian National Research Centre on Aging (IRCCS INRCA), Contrada Mossa, 2, 63900 Fermo, Italy; 2 Geriatric-Rehabilitative Department, Italian National Research Centre on Aging (IRCCS INRCA), Contrada Mossa, Fermo, Italy; 3 Center for Neurobiology of Aging, (IRCCS INRCA), Via Birarelli, Ancona, Italy; 4 Unit of Geriatric Pharmacoepidemiology and Biostatistics, Italian National Research Centre on Aging (IRCCS INRCA), Via S. Margherita, Ancona, Italy; 5 Advanced Technology Center for Aging Research, Scientific Technological Area, Italian National Research Centre on Aging (IRCCS INRCA), Via Birarelli, Ancona, Italy

**Keywords:** Mild cognitive impairment, Biomarkers, Comprehensive intervention, Adapted tango, Older adults

## Abstract

**Background:**

Dementia represents a key health issue for older adults, with negative consequences on psycho-social and functional status. Treatments that counteract cognitive deficits in mild cognitive impairment (MCI) are needed to prevent or delay it.

**Aim:**

To describe the experimental protocol of the STRENGTH Project. This study investigates a multimodal intervention in older adults with MCI to improve cognitive, functional, biochemical and psycho-social aspects.

**Methods:**

The prospective randomised controlled trial will enrol 300 subjects with MCI (age ≥ 60 years). Participants will be randomly assigned to: (a) the experimental group, which will undergo sessions of adapted tango, music therapy, engagement in social activities, cognitive intervention and psycho-education for 6 months or (b) the control group, which will receive psycho-education and advice on healthy lifestyle for 6 months. All outcomes will be analysed before intervention (baseline), immediately after termination (follow-up 1), after 6 months (follow-up 2) and after 2 years (follow-up 3).

**Discussion:**

We expect that the findings of this multidisciplinary study will be useful to optimize clinical and psycho-social interventions for improving cognitive and functional status of subjects with MCI.

**Conclusions:**

This project could have a meaningful impact on National Health Systems by providing clues on multidisciplinary management of older adults affected by cognitive decline to prevent dementia.

## Introduction

Dementia is a chronic and progressive syndrome that affects 5–8% of general population aged 60 years or more [1]. Dementia damages memory, thinking and behaviour and has negative consequences on psycho-social functioning. Treatments that counteract cognitive deficits in mild cognitive impairment (MCI), a translational state between age-associated cognitive decline and diagnosed dementia, are needed to prevent or delay it.

Previous studies evidenced that currently available pharmacological therapies are not effective [2], while the use of psycho-social interventions in association with cognitive training and physical activity (PA) has been recognized to improve functional capabilities of older adults, thus positively acting on the onset of neurological diseases [3]. Management of cardiovascular and lifestyle-related risk factors is particularly promising [4]. Healthy lifestyle, engagement in regular moderate PA, and involvement in social recreational activities may have protective effects both on mental functions and on risk of dementia [5–7]. Data from our previous "My Mind Project" (Grant no. 154/GR-2009–1584108) showed that subjects affected by MCI had lower level of PA and social participation than age-matched healthy controls and highlighted the potential negative consequences of this situation on psychological conditions and functional status [8]. These findings suggest the necessity to develop multimodal interventions for elderly people with compromised cognition [9]. Indeed, according to the literature [3, 10], single-domain trials did not yield long-term effects and, consequently, are not really effective for dementia prevention. Thus, in the STRENGTH Project, we decided to study the effect of a multimodal intervention that associates PA (i.e., adapted tango for non-demented elderly people), cognitive stimulation, music therapy and other psycho-social treatments.

PA programmes based on dance were proved to promote in the elderly population the preservation of functional mobility, the improvement of cognitive processes, mood status and well-being and reduction in the fear of falling [11–13]. In particular, a randomised, controlled trial conducted in Italy evidenced specific psycho-social and physical benefits in applying PA based on dance, thanks to the improved balance capability and the reduced risk of falling in the enrolled elders [14]. Nevertheless, more studies regarding dance interventions in the Italian elderly population are needed [11].

Adapted tango was previously tested by other authors, in subjects with different cognitive status, as it was proven to be feasible and safe for improving mobility, physical functions, spatial cognition and memory with maintenance of the positive achievements for 3 months after the end of the intervention [15, 16]. Moreover, it seems that tango is a particularly innovative approach because of its low PA impact and its effectiveness in promoting positive emotional state and alleviating psychological distress, such as anxiety and depression status in elderly people [17–19]. Depression, anxiety and psychological stress are often observed in MCI subjects due to their consciousness of the cognitive decline; moreover, their subjective memory complaints could intensify the deficit perception worsening their performances [9].

Given this background, the aim of our study is to ameliorate cognition, physical functioning and quality of life in MCI patients, with a potential positive impact on cognitive decline and dementia onset prevention/delay.

## Methods

### Study design

**Fig. 1:**
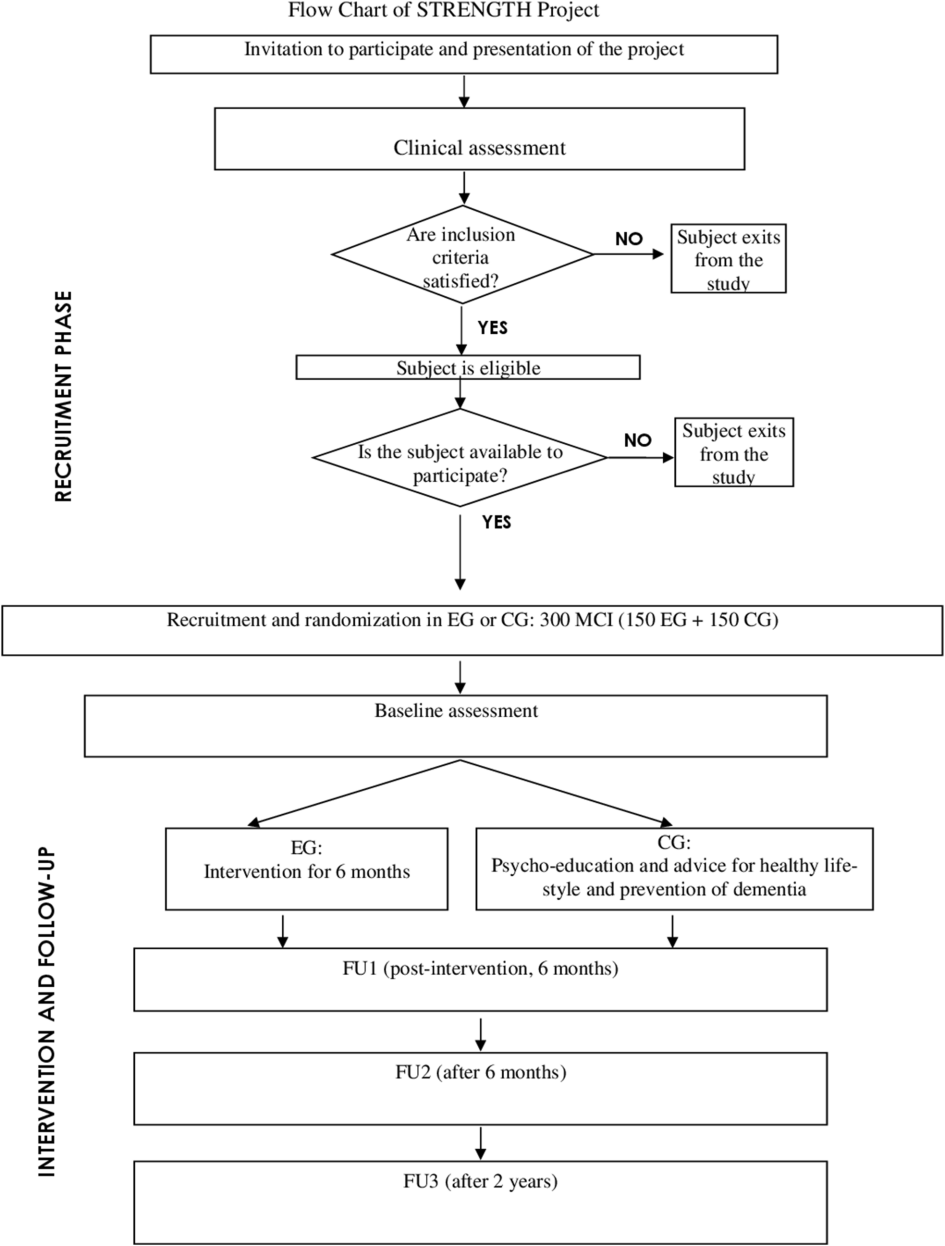
Flowchart of strength project

The STRENGTH Project is a prospective randomised controlled trial for the assessment of the effects of a multimodal intervention (i.e., adapted tango, music therapy, engagement in social activities, cognitive stimulation, and psycho-education for 6 months) in subjects with MCI. The study uses a multidisciplinary approach, has a 3-year duration and includes 3 follow-up phases, as indicated in Fig. 1.

#### Specific aim 1

To study the effect of the multimodal intervention on cognitive performances, functional status, and mobility in MCI subjects by objectively measured parameters (baseline vs. follow-up 1, follow-up 2 and follow-up 3). Mobility (i.e., the ability of a subject to move in his/her environment) is often compromised in elderly people as a consequence of falls and other injuries and this could cause disability and reduced social engagement. The identification of approaches that might prevent motor–cognitive impairment would be a pivotal advancement in the correct management of aging people. Mobility will be evaluated by means of balance, walking test and gait speed; motor–cognitive function, defined as a process of coordination of movements finalized to a particular motor goal, will be measured by means of visual–spatial memory task.

The longitudinal monitoring of cognitive decline and dementia onset will also be implemented (follow-up 2 and follow-up 3).

#### Specific aim 2

To study the effect of the intervention on psycho-social aspects (e.g., psychological well-being, perceived stress, quality of life, metacognition and mood status), lifestyle characteristics and social network (baseline vs. follow-up 1, follow-up 2 and follow-up 3).

#### Specific aim 3

To study the effect of the intervention on specific biomarkers (baseline vs. follow-up 1, follow-up 2 and follow-up 3).

#### Experimental design aim 1

Neuropsychological as well as cognitive and clinical assessment, including cardiovascular, anthropometric, and functional evaluation (e.g., blood pressure, body mass index, ultrasound heart examination through standard echocardiogram, 6-min walk test, gait speed, Berg Balance Scale, handgrip strength, Physical Activity Scale for the Elderly, basic activities of daily living and instrumental activities of daily living) will be carried out (Table 1).

**Table 1:** Clinical, functional, and neuropsychological assessment

Instruments
*Cognitive assessment*
Clinical Dementia Rating Scale—CDR [20]
Memory Complaint Questionnaire—MAC-Q [21]
Montreal cognitive assessment—MoCA [22]
Mini mental state examination—MMSE [23]
Rey auditory verbal learning test—RAVLT [24]
Phonological verbal fluency—PVF [24]
Supra-span of Corsi [25]
Semantic verbal fluency—SVF [26]
Attentive matrices [26]
Trail making test A−B—TMT A–B [27]
*Psychological assessment*
Depression Anxiety Stress Scale—DASS [28, 29]
Geriatric Depression Scale-15—GDS-15 [30]
Psychological Well-Being Scales—PWB [31, 32]
SF-36 (36 health survey) [33]
*Functional assessment*
Basic activities of daily living—ADL [34]
Instrumental activities of daily living—IADL [35]
Berg Balance Scale—BBS [36]
6-min walking test—6MWT [37]
*Social network*
Lubben Social Network Scale—LSNS [38]
*Lifestyle characteristics and physical activity*
Physical Activity Scale for the Elderly—PASE [39]
Lifestyle Questionnaire

#### Experimental design aim 2

Enrolled subjects will undergo a detailed protocol of tests and questionnaires to evaluate the following aspects: psychological well-being, perceived stress, quality of life, metacognition, mood status, lifestyle characteristics and social network (Table 1) [20–39].

#### Experimental design aim 3

**Fig. 2:**
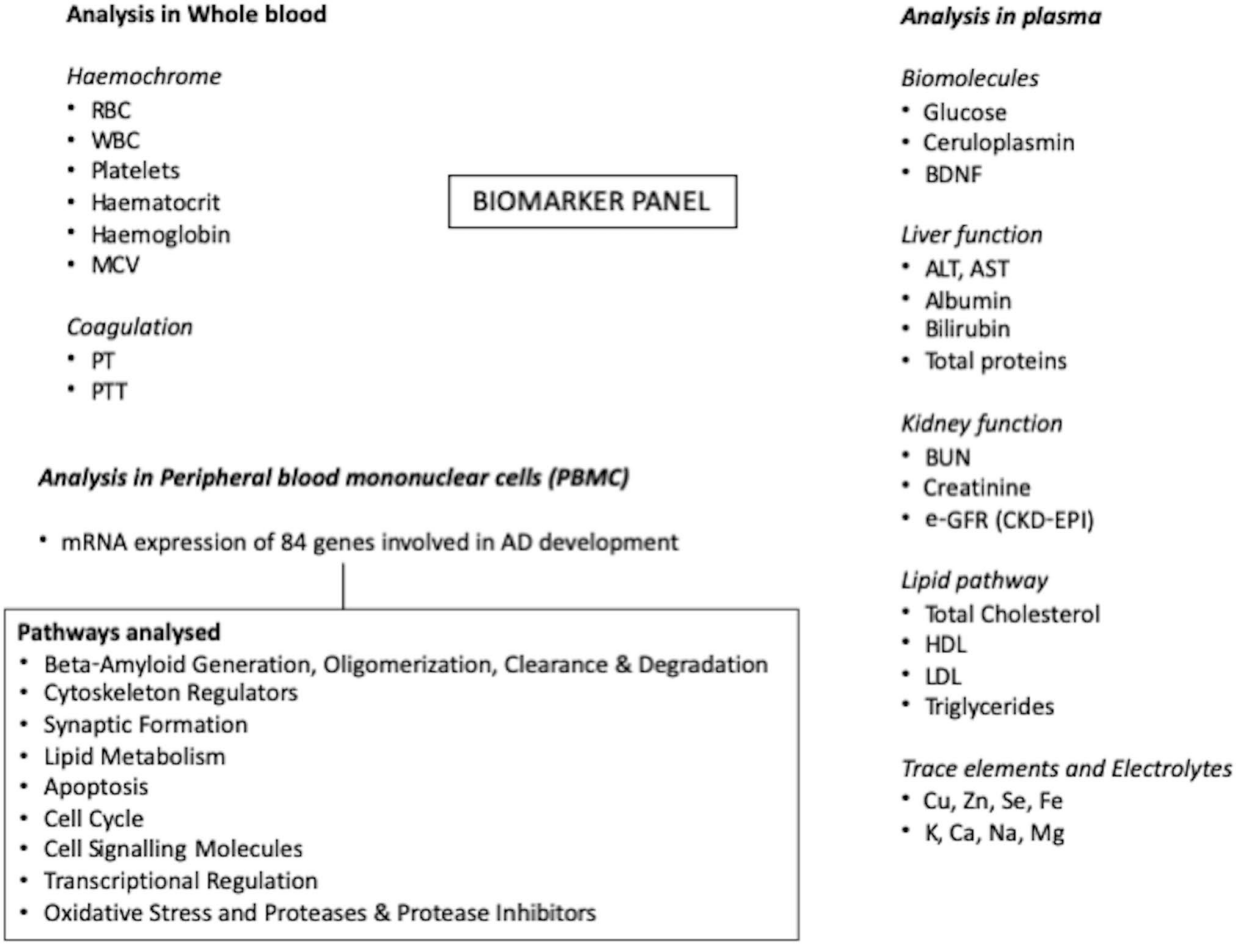
The panel represents all the biomarkers that will be analysed in the STRENGTH Project for each tissue (i.e., whole blood, plasma, and peripheral blood mononuclear cells). *RBC* red blood cells, *WBC* white blood cells, *MCV* mean corpuscular volume, *PT* prothrombin time, *PTT* partial thromboplastin time, *BDNF* brain-derived neurotrophic factor, *ALT* alanine amino transferase, *AST* aspartate amino transferase, *BUN* blood urea nitrogen, *e-GFR* estimated glomerular filtration rate, *CKD-EPI* chronic kidney disease epidemiology collaboration, *HDL* high-density lipoprotein, *LDL* low-density lipoprotein, *Cu* copper, *Zn* Zinc, *Se* selenium, *Fe* iron, *Ca* calcium, *Mg* magnesium, *Na* sodium, *K* potassium, *AD* Alzheimer’s Disease

The following parameters will be evaluated (details are reported in Fig. 2):

- BDNF and ceruloplasmin protein amounts in plasma and their mRNA expression in peripheral blood monuclear cells (PBMC), as the role of peripheral leukocyte dysfunctions in Alzheimer’s disease (AD) development [40, 41] is well known;
- mRNA expression in PBMC of 84 key genes involved in AD physiopathology (i.e., beta-amyloid generation, oligomerization, clearance, and degradation; cytoskeleton regulators; synaptic formation; lipid metabolism; apoptosis; cell cycle; cell signalling molecules; transcriptional regulation; oxidative stress; proteases and protease inhibitors);
- plasma copper, zinc, and other important micronutrients;
- a panel of the most common laboratory parameters.

### Recruitment and screening phase

The project started on 1 August 2019 and will last until 31 July 2022. The protocol was approved by the local ethics committee (Bioethics Advisory Committee of IRCCS INRCA, Ancona, Italy; code no. 18006). Three hundred community-dwelling older adults (age ≥ 60 years) with diagnosis of MCI will be enrolled in the Evaluation of dementia’s disease Unit of IRCCS INRCA Hospital in Fermo. MCI will be diagnosed by an extended neuropsychological, clinical and functional evaluation, as well as by neuroimaging and laboratory tests according to diagnostic guidelines [42]. The subjects’ screening will be performed during routine clinical visits applying specific inclusion/exclusion criteria as explicated below. Written informed consent will be obtained from all subjects.

### Randomisation

The selected subjects will be randomly allocated to the experimental group (EG, *n* = 150) or to the control group (CG, *n* = 150) using a randomisation list.

### Inclusion and exclusion criteria

The inclusion criteria comprise: (a) diagnosis of MCI according to the guidelines of the National Institute of Aging [42]; (b) age 60 years or older, (c) availability during the intervention and testing phases, (d) presence of a caregiver; (e) capability to sign the informed consent.

Exclusion criteria include: (a) presence of sensory-physical deficits that would prevent participation in the study; (b) diagnosis of dementia; (c) presence of serious medical and/or psychiatric conditions.

### Sample size calculation

It was based on the null hypothesis that proportion of “success” and “failure” of the intervention is equal in the two groups (i.e., EG and CG), such as OR = 1.

Success and failure were defined on the basis of significant variation in the primary outcomes. Considering an OR = 1.5 indicative of an effect of the intervention, a total sample of 300 subjects allows us to reach a 90% of statistical power using logistic regression at the 5% level (two tailed). Drop-out rate was set at 20%.

### Intervention

A group approach will be used. The intervention will be focused not only on cognitive enhancement, but also on several other aspects, such as advice and psycho-education about healthy lifestyle strategies as well as engagement in leisure activities. EG will be followed by a multidisciplinary team (i.e., psychologists, physicians, nurses, tango instructors and music therapist).

Multimodal intervention will consist in a 20-class syllabus of 180-min session, once a week, including 90 min of adapted tango in accordance to guidelines for physical activity in aging and literature [15], and, in the same session, further 90 min of comprehensive cognitive intervention for 6 months. The intervention will maximize social participation. Tango will be adapted for older adults with mild cognitive decline and possible mild balance and musculoskeletal impairment. Tango classes will be carried out in a ballroom dancing, led by two experienced and qualified Argentine instructors, with years of certified proficiency and experience in teaching adapted tango methods to elderly people. Classes will take into account the engagement of participants and feasibility. Each class will include about 25 subjects who will dance in pairs with other participants, including instructors, relatives and caregivers. Classes will begin with a phase of warm-up consisting of postural alignment and breathing. The main phase of dance will include enhancement exercises and addition of novel simple steps at each class with varied speeds and rhythms, individually and in pairs, focused on body awareness to educate subjects on prevention of falls, improving mobility, foot placement, whole body coordination, postural control, balance, self-confidence and attention to dance partner. In the last phase, participants can share their experience or ask questions. Possible adverse events will be tracked.

Cognitive intervention will include restorative and compensatory cognitive stimulation, learning mnemonics strategies and music therapy, and will be carried out by two trained psychologists and a music therapist. Intervention will use a metacognitive and a motivational approach applied to subjects with MCI, as described in previous studies [43, 44]. Participants will be also required to perform home exercises, which will include some cognitive stimulation exercises, assigned at the end of each class. Homework will be reviewed by the psychologist during the next class.

An example syllabus of classes is reported in Table 2.

**Table 2:** Example syllabus of classes

Classes			
First class	Welcome to participants. Subjects can share their experience about cognitive impairment or ask questions.	75 min of psycho-education about healthy lifestyle aimed to prevention of dementia and other pathologies	90 min of dance. Explanation about the aim of adapted tango
Second class	Welcome to participants. Questions of participants.	75 min of psycho-education and health advice about the cognitive enhancement for prevention and management of cognitive disorders. Assignment of homework	90 min of adapted tango, as indicated in “Methodology”
Third class	Welcome to participants. Questions of participants. Revision of homework assigned the previous week.	75 min of cognitive stimulation aimed to learning of mnemonic strategies. Assignment of homework	90 min of adapted tango, as indicated in “Methodology”
Fourth class	Welcome to participants. Questions of participants. Revision of homework assigned the previous week.	75 min of music therapy. Assignment of homework	90 min of adapted tango, as indicated in “Methodology”

Possible adverse events, adherence to the study and participants’ attrition will be monitored by the principal investigator, adapted tango teachers and other staff members involved in the intervention.

To avoid bias effect of the intervention, GC will receive psycho-education and advice for healthy lifestyle as well as suggestions about the prevention of dementia once a month for 6 months. This group will receive the same clinical monitoring of the general health status, neuropsychological assessment, and cardiovascular parameters of EG. Moreover, they will be informed about the results and possible benefits of intervention, so they could have the possibility to start classes of tango therapy at the end of the study, if they so wish. Indeed, at the end of the study, we expect that this type of program will become a new service offered by IRCCS INRCA to elderly persons with an increased risk of cognitive decline.

#### Primary outcome: success of the intervention

The response to the intervention will be evaluated using performance in the Corsi test [25]: an increase of at least 1 point will indicate a success based on the enhancement of the spatial learning processes.

#### Secondary outcomes

They include:

- the assessment of the intervention effects on lifestyle characteristics as well as psychological, functional and health status;
- the assessment of the intervention effects on mobility;
- the investigation of the conversion rate from MCI to dementia;
- the identification of biomarkers able to mirror the intervention effects.

### Statistical analysis

All data will be collected in a database. A descriptive analysis will be performed to assess the distribution of variables and the presence of possible outliers. The latter will be checked and identified separately and then excluded from the analysis. For normally distributed variables, the comparisons between EG and CG at baseline will be carried out by *t *Student test or Chi-square test, while those between baseline and follow-up phases by paired sample *t* test. For non-normally distributed variables, non-parametric tests will be used. Correlation analysis will be performed to evaluate the relationships among variables and general linear models and repeated measures ANOVA will be used to assess the intervention effects adjusted for confounding variables (e.g., gender, age classes, marital status, schooling, drugs, and other variables that will emerge after analysis of secondary outcomes). The SPSS software will be employed.

## Discussion and conclusion

The main expected result is the development of a clinical and psycho-social intervention that is able to reduce age-related disability (both physical and cognitive), improve quality of life, well-being and socialization, and counteract cognitive decline and dementia development. The treatment will include different domains, such as rehabilitation and enhancing therapy, monitoring of health status and cardiovascular parameters, psycho-education and neuropsychological stimulation. Socio-relational aspects with a positive impact on psychological status will also be taken into account.

The innovative topic is the use of adapted tango in our multimodal intervention: it seems to enhance physical-cognitive aspects because of the required alternation of attention among postural control, current balance, cognition-challenging step, and spatial memory. In particular, previous studies have evidenced that this kind of dance is sustainable and acceptable by independent elderly people, because it is a feasible PA [13]. Benefits were observed in activities of daily living (ADL) performance and quality of life of older adults [15] and, its benefits known, it was successfully applied to patients with different neurological conditions [16, 45]. A recent paper evidenced that tango has many favourable therapeutic characteristics, but it is important to check and evaluate the progress and the adverse events during the intervention to prevent side effects as well as to optimize tolerance and adherence [18]. Few studies described the occurrence of adverse events or untoward results, so they will be monitored. Classes will be well-structured, to promote socialization, feasibility, and engagement. Therefore, according to the patient’s status, this therapeutic intervention should be built by a multidisciplinary team, which is going to include clinical staff, in accordance to medical concept [18]. In our study, an important achievement will be the creation of a multidisciplinary, highly specialized team of professionals who will work together to improve the quality of life of the older adults affected by MCI. These subjects at high risk of developing dementia could be treated early with a focused intervention, to slow the rate of conversion to dementia or prevent the conversion. Moreover, the evaluation of functional aspects, not only in terms of preservation of functional independence in daily life activities, but also in terms of reduction of falls, enhanced capacity to walk/move, maintain gait speed and balance without or with minimal assistance, and improvement of cognitive and psycho-social functions, will be used to identify the benefits of intervention at the end of the 3 years.

We will deepen the consequences on community-dwelling older subjects with MCI and will test a longer protocol (i.e., 6 months) not previously considered. Another key element is the evaluation of engagement in psycho-social and relational activities related not only to dance but also to cognitive stimulation, music therapy included.

The multidisciplinary approach will be strengthened by the evaluation of several biomarkers: this could help us better define the intervention efficacy. Indeed, in a previous study (i.e., My Mind Project), this approach allowed operators to display the positive effect of a comprehensive intervention based on cognitive stimulation on specific biological dysfunctions due to MCI or AD, such as platelet total phospholipases A2 activity [46], lymphocytic brain-derived neurotrophic factor (BDNF) mRNA expression [47], as well as plasma BDNF concentration [48].

This project might have a significant impact on National Health Systems by providing a multidisciplinary management of MCI subjects to slow down cognitive decline and potentially reduce the conversion rate to dementia. In Italy and worldwide, dementia represents a pivotal public health issue that negatively affects older persons’ independence, thus creating medical, social, and economic burdens. It is essential to define specific interventions to increase quality of life and well-being in the elderly: such type of programs could become a new service offered by the health services to older adults.
